# Directivity Dependence of a Distributed Fiber Optic Hydrophone on Array Structure

**DOI:** 10.3390/s22166297

**Published:** 2022-08-21

**Authors:** Wenmin Li, Yu Chen, Yan Liang, Yang Lu, Zhou Meng

**Affiliations:** College of Meteorology and Oceanography, National University of Defense Technology, Changsha 410073, China

**Keywords:** distributed fiber optic hydrophone, directivity function, channel length, channel spacing

## Abstract

A distributed fiber optic hydrophone (DFOH) is a new type of fiber optic hydrophone (FOH) with adjustable structure. The dependence of the directivity of a DFOH on array structure is theoretically and experimentally studied. The directivity function of a sensing channel and that of a DFOH are derived. Based on the directivity function, the simulations are performed. Finally, the theoretical analysis is demonstrated by the experiments performed on Qingyang lake, and the results reveal that the longer sensing channel length guarantees the lower first-order side lobe and the narrower main lobe. As the channel length increased from 1 to 3, the main lobe width and first-order side lobe height decreased by 4.9° and 6 dB, respectively. In addition, channel spacing is irrelevant to directivity as the spacing is shorter than the wavelength. As the channel spacing increased from 0 to 1, the variations of the main lobe width and first-order side lobe height are lower than 0.5° and 0.94 dB, respectively. This study would provide guidance for the structure design of a distributed fiber optic hydrophone in signal processing.

## 1. Introduction

**Figure 1:**
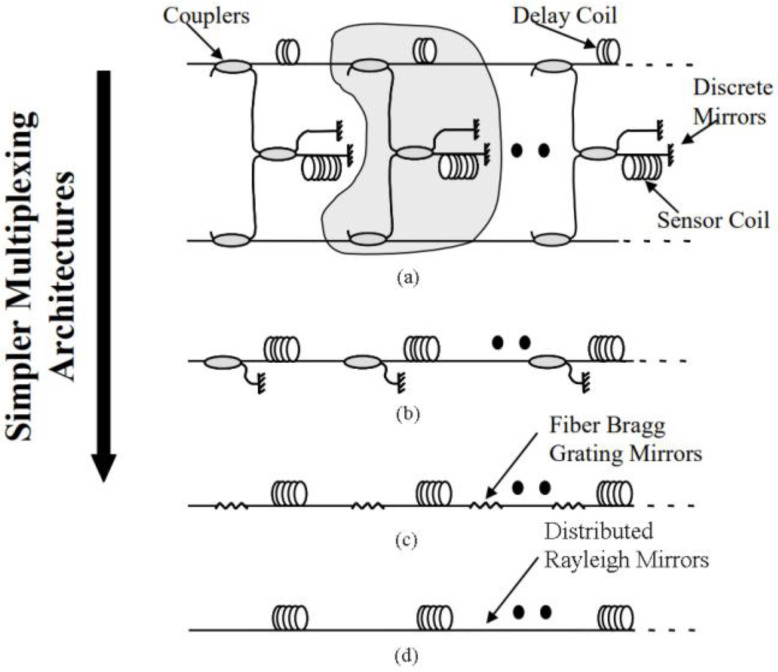
The development history of the structure of the FOH: (**a**) Conventional TDM, FDM architectures; (**b**) Inline Michelson; (**c**) Low Finesse Fabre-Perot; (**d**) Distributed fiber optic hydrophone.

Distributed acoustic sensing (DAS) is an attractive fiber optic sensing technology for spatially continuous acoustic signal measurements over long distances. By using a wide range of optical cables, various vibration sources in the surrounding environment can be sensed with high sensitivity and located precisely [1,2,3,4]. DAS has been widely employed in many fields such as railway transportation [5,6], perimeter security [7], pipeline security [8,9], etc. Applying DAS technology to the field of underwater acoustic sensing gives birth to a new type of fiber-optic hydrophone (FOH), distributed fiber optic hydrophone (DFOH). Conventional FOHs are point sensors and need to be formed into arrays to detect acoustic signals [10,11,12,13]. A DFOH functions as an FOH array and interrogates acoustic signals utilizing DAS technology. Compared to a conventional FOH array, a DFOH has some unique advantages. Firstly, it can pick up underwater acoustic signals continuously in space thanks to the DAS technology. Secondly, as shown at the bottom of Figure 1 [14], a DFOH is basically comprised of only fiber. However, as shown at the top of Figure 1, a conventional FOH consists of many fiber optic components such as fiber couplers and Faraday rotation mirrors. A simple structure of a DFOH makes it more reliable than a conventional FOH.

A FOH array is designed to orientate acoustic signals, and its directivity is specified by usage of a directivity function. The width of the main lobe and the height of the side lobe are two key parameters of a directivity function, and they are expected uniquely in different practical use. The two parameters are determined by the array structure of a FOH array such as the length of a sensing channel and the space between two adjacent channels. For a conventional FOH array, the array structure is fixed. Accordingly, the directivity function of the array is unique, which limits the array in widely practical use. Benefiting from DAS technology, the sensing channel length and sensing channel space of a DFOH are set in signal processing and thus are changeable. This makes a DFOH perform changeable directivity and be applicable to various practical use. To meet the directivity requirement in a particular application, the array structure of a DFOH has to be carefully designed in signal processing, and the dependence of the directivity function on the array structure of a DFOH is expected firstly. Currently, there are few studies reported on the dependence of the directivity function on the array structure of a DFOH. Lu et al. developed a DFOH and analyzed its performance [15]. Only the response of a sensing channel on the orientation of detected acoustic signals was theoretically analyzed, and the directivity function of the DFOH was not studied. Besides, no experiments were performed to verify the theoretical analysis.

This paper focuses on the dependence of directivity function on the array structure of a DFOH. The directivity function of a sensing channel and that of a DFOH are derived. Based on the directivity function, the simulations are performed to theoretically analyze the dependence of the array directivity function on the array structure. Finally, the theoretical analysis is verified by the experiments. In the experiment, a DFOH system including a DFOH and a DAS system based on phase-sensitive optical time-domain reflectometry (Φ-OTDR) is utilized [16].

## 2. Directivity Function of a DFOH

We shall firstly define the structural parameters of a DFOH. Typically, a DFOH is comprised of a sensing fiber wrapped continuously around an elastic cylinder at a certain wrapping ratio R. Here, the ratio R is defined as the ratio of the length of a wrapped fiber to the length of the elastic cylinder. On the signal processing end, a DFOH is virtually divided into a sequence of discrete sensing channels in tandem with identical space d, and the number of the channels is N. The length of a sensing channel is *L* = *G*/*R*, where *G* refers to the gauge length in a DAS system. For a DAS system that interrogates a phase of Rayleigh backscattering light-waves (RBLs) along a sensing fiber [17,18], *G* can be specified arbitrarily. As a result, the length L of a sensing channel is adjustable. Channel spacing *d* can be shorter than the length of a sensing channel L since the channels are virtually divided and initial points can be selected arbitrarily. In other words, two adjustable sensing channels can overlap each other, which is impossible for a conventional FOH array.

### 2.1. Directivity Function of a Sensing Channel

**Figure 2:**
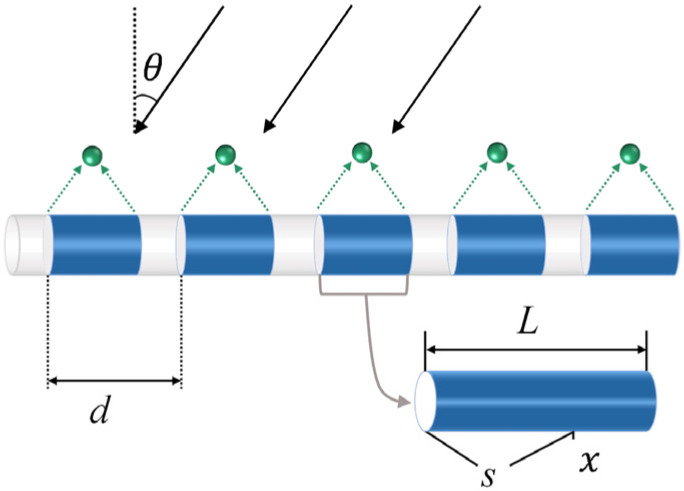
Array structure and acoustic signal receiving model of a DFOH.

A sensing channel in a DFOH transduces external acoustic signals to the phase change of a Rayleigh backscattered light (RBL). As shown in Figure 2, *L* is the length of a sensing channel, *x* is a reference position in the channel, and s is the distance between *x* and the initial point of the channel [19,20]. As a far-field acoustic signal orientated at an angle *θ* to the radial direction of a sensing channel is imposed on the sensing fiber wrapped on the channel, the refractive index of the fiber at position *x* of the sensing channel is changed by

(1) $$\Delta n\left(x\right)=P\eta \cos\left(\frac{2\pi }{\lambda }\sin\left(\theta \right)x-\omega t\right)$$

where P, *ω*, and *λ* are the sound pressure, angler frequency, and the wavelength of the acoustic signal, respectively, and *η* is a composite response coefficient [21,22]. Accordingly, the phase change as the output of the sensing channel is calculated as [23,24,25]

(2) $$\Delta \phi =2\int_{x-s}^{x+\left(L-s\right)}\frac{2\pi }{\lambda_{l}}\Delta n\left(x\right)dx$$

where *λ_l_* is the wavelength of a light-wave propagating in the sensing fiber, and the coefficient 2 arises from the round trip that a light-wave undergoes. Applying Equation (1) to Equation (2) gives (see Appendix A for the detailed derivation)

(3) $$\Delta \phi =\Delta \phi_{0}\cos\left[\frac{2\pi }{\lambda }\sin\left(\theta \right)x+\varphi_{i}-\omega t\right]$$

where $\varphi_{i}=2\pi \sin\left(\theta \right)\left(0.5L-s\right)/\lambda$, $\Delta \phi_{0}=4\pi P\eta LB_{1}/\lambda_{l}$ is the amplitude of the output phase Δϕ, and $B_{1}$ is a coefficient and is expressed as

(4) $$B_{1}=\sin c(\frac{L}{\lambda }\sin\theta )$$

Equation (3) indicates that the output phase Δϕ of a sensing channel oscillates at the identical frequency to the acoustic signal with a phase delay $\varphi_{i}$ determined by s, L, and *θ*. In addition, the amplitude of the phase is governed by the coefficient *B*_1_. As Equation (4) reveals, $B_{1}$ varies with *θ*. As a result, the amplitude of the output phase Δϕ becomes directive, and this directivity is more obvious as the ratio L/λ becomes larger. Considering the dependence of *B*_1_ on *θ*, *B*_1_ is defined as the directivity function of a sensing channel.

Essentially, a sensing channel is equivalent to a sequence of point sensors continuously distributing along the sensing channel with identical spacing. Because the acoustic signal arrives at the sensors at a different time, there exists a constant phase delay δ*φ* between the output phase of two adjacent sensors, and δ*φ* various with *θ*. The output phase Δϕ of a sensing channel is the sum of the output phase of all the point sensors, and its amplitude is determined by δ*φ*. Since δ*φ* varies with *θ*, the amplitude $\Delta \phi_{0}$ becomes directive. As the ratio L/λ increases, the phase delay between the output phase of two points sensors located at two ends of the sensing channel increases, and thus the amplitude of $\Delta \phi_{0}$ becomes more directive.

### 2.2. Directivity Function of a DFOH

The directivity of a DFOH results from the phase delay between two adjacent sensing channels. In order to eliminate the impact of the phase delay $\varphi_{i}$ in Equation (3) on the directivity of a DFOH, the reference positions of all the sensing channels in the DFOH are selected at the identical position relative to their own initial points. For simplification, s=0.5L is set in Equation (3). Accordingly, $\varphi_{i}=0$ is obtained, and Equation (3) is reduced to

(5) $$\Delta \phi =\Delta \phi_{0}\cos\left[\frac{2\pi }{\lambda }\sin\left(\theta \right)x-\omega t\right]$$

To obtain the directivity function of a DFOH, a DFOH is considered as a linear discrete FOH array consisting of a sequence of discrete sensing channels in tandem with identical space d, and the number of the channels is N. Adding up the output phase changes of all the sensing channels leads to an output phase of a DFOH. The normalized amplitude of the output phase is the directivity function of the DFOH, and is given by:

(6) $$\begin{array}{cc} B & =|\sin c(\frac{L}{\lambda }\sin\theta )\cdot \frac{\sin(N\frac{d\pi }{\lambda }\sin\theta )}{N\sin(\frac{d\pi }{\lambda }\sin\theta )}|\\ & =B_{1}B_{2} \end{array}$$

Equation (6) indicates that the directivity function of a DFOH is the product of the directivity functions of a sensing channel and a conventional linear discrete FOH array. In addition, Equation (6) reveals that the directivity function of a DFOH is dependent on the structure of a DFOH such as the channel length L and channel spacing d.

## 3. Directivity Dependence of a DFOH on Array Structure Parameters

For a well-developed FOH array, the directivity function of the array is fixed since the channel length and channel spacing cannot be changed. In comparison, the channel length and channel spacing of a DFOH are adjustable, which makes it possible to desire the directivity function of a DFOH even if the DFOH is well developed. In this section, the simulation proceeds based on Equation (4) to analyze the effects of channel length and channel spacing of a DFOH on the directivity function, especially focusing on the effects on the main lobe and side lobe of the directivity function. Considering the fact that the total length of the well-developed DFOH is fixed, the total length of the DFOH is set as a constant in the following analysis. Besides the structure of a DFOH, the directivity function is also determined by the wavelength of an acoustic signal, as Equation (4) indicates. In order to study the universal law applicable to acoustic signals of all wavelengths, rather than the law only for a particular wavelength, the channel length and the channel spacing are normalized as L/λ and d/λ, respectively.

### 3.1. Channel Length

**Figure 3:**
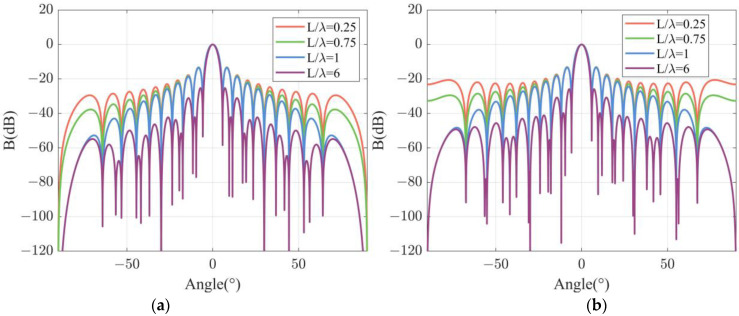
Simulated beam patterns for different channel lengths at specific channel spacing: (**a**) d/λ=0.25; (**b**) d/λ=0.75.

The dependence of directivity function *B* on L/λ is analyzed firstly. In the simulation, the total length of a DFOH is set as dN/λ=10 with N being the number of sensing channels. In the case of d/λ=0.25, four directivity functions are simulated and are presented in Figure 3a as L/λ are set as 0.25, 0.75, 1, and 6. The widths of the main lobes and the heights of the first side lobes are shown in Table 1. The simulation results show that as L/λ increases from 0.25 to 6, the widths of the main lobes are 5.04°, 5.04°, 5.04°, and 4.41°, respectively. The widths of the main lobes remain stable in the case of L/λ<1 and decrease in the case of L/λ>1. In addition, the height of the side lobes at the same angle decreases as L/λ increases, and it decreases more dramatically at larger angles. The heights of the first side lobes are −13.26 dB, −13.41 dB, −13.54 dB, and −25.31 dB, respectively. The conclusions mentioned above are also valid in the cases of d/λ = 0.75, which are confirmed by the simulation results shown in Figure 3b and Table 2.

The dependence of directivity function *B* on the channel length analyzed above originates from the directive response of a sensing channel *B*_1_ to external acoustic signals. As Equation (2) indicates, the response of a sensing channel is more directive as the sensing channel becomes longer, leading to more of a directive response from a DFOH.

### 3.2. Channel Spacing

**Figure 4:**
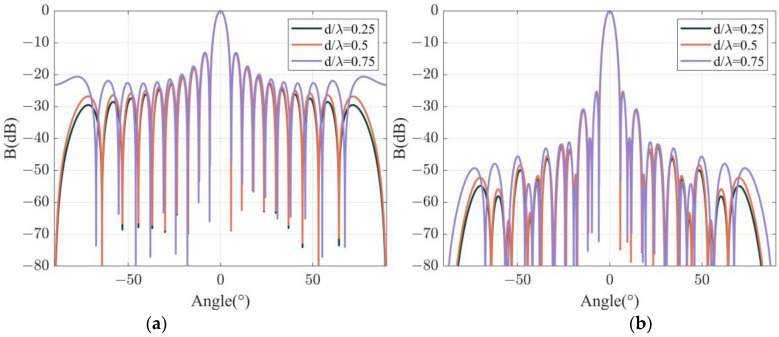
Simulated beam patterns for different channel spacings at specific channel length: (**a**) L/λ=0.25; (**b**)L/λ=6.

The effect of channel spacing is analyzed in this section. The total length of a DFOH is set to dN/λ=10 as in Section 3.1. When L/λ=0.25, three directivity functions are simulated and are shown in Figure 4a as d/λ is set as 0.25, 0.5, and 0.75. The widths of the main lobes and the heights of the first side lobes are shown in Table 3. The simulation results show that when d/λ increases, the widths of the main lobes are 5.04°, 5.04°, and 5.21°, respectively. Additionally, the heights of the first side lobes are −13.26 dB, −13.21 dB, and −13.11 dB, respectively. Both the main lobes and the first side lobes change slightly in the above simulation, and it is also valid when L/λ = 6, which is shown in Figure 4b and Table 4. Specifically, when L/λ = 6, the widths of the main lobes are 4.41°, 4.41°, and 4.47°, respectively. Additionally, the heights of the first side lobes are −25.31 dB, −25.27 dB, and −25.93 dB, respectively.

The dependence of directivity function *B* on the channel spacing analyzed above only covered the case of d/λ<1, and this is because grating lobes will appear when d/λ>1. In time domain signal sampling, a similar phenomenon occurs when the sampling period is larger than the signal period. Therefore, it is important to avoid d/λ<1 in the DFOH array.

## 4. Experiment Verification

Experiments are performed on Qingyang lake using a self-developed DFOH system including a DFOH of 8 m and a DAS system. The DFOH consists of a sensing fiber wrapped uniformly around an elastic cylinder at a wrapping ratio *R* of 10. The DAS system employed in the experiment is self-developed, and the details of the system are introduced in [16]. Probe pulses of 50 ns pulse duration interrogate the DFOH at a rate of 100 kHz. In addition, the sampling rate of the DAS system is $f_{DAQ}=250$ Mbps. Therefore, the minimum channel spacing in fiber length is $d_{f}=c/2nf_{DAQ}=0.4$ m, where *c* is the speed of light in vacuum, and *n* is the group refractive index. Accordingly, the minimum channel spacing in a DFOH is $d_{\min}=d_{f}/R=0.04$ m. The channel length is specified in signal processing by the design of gauge length such that L=G/R is satisfied. During the experiments, the acoustic signal oscillating at 1000 Hz (corresponding to a wavelength of 1.5 m) is emitted from an acoustic source fixed at a position 5 m beneath the surface of the lake.

**Figure 5:**
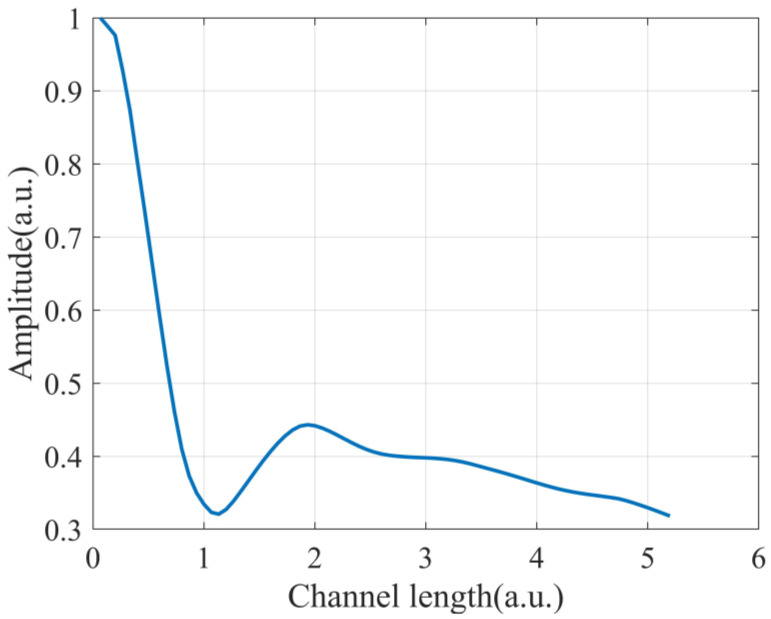
Measured amplitude of Δϕ as a function of channel length.

Firstly, directivity dependence of a sensing channel on channel length L is experimentally verified. The output of a sensing channel is a time-varying phase Δϕ oscillating at 1000 Hz, and the amplitude of Δϕ is linearly proportional to directivity function *B*_1_ for a certain L, *θ,* and *λ*. During signal processing, channel length L is changed, and the amplitude of Δϕ as a function of L is presented in Figure 5. The amplitude for each channel length L in Figure 5 is the average value of phase amplitudes of ten contiguous channels spaced by $d_{\min}=0.04$ m. The results show that the phase amplitude varies with L in the way of a sinc function. Since the amplitude of Δϕ is linearly proportional to *B*_1_, the results in Figure 5 confirm *B*_1_ as a sinc function of L, which is consistent with Equation (4).

**Figure 6:**
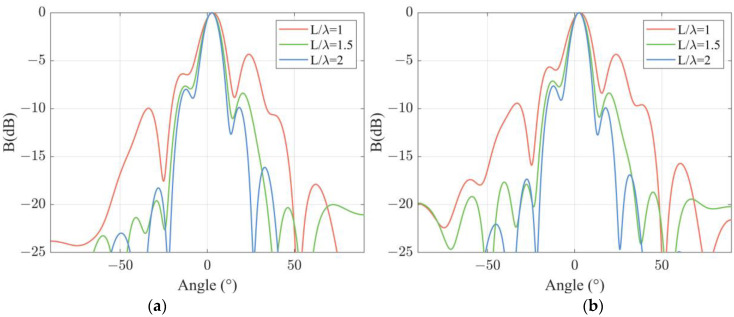
Experimental results of beam patterns at different channel lengths at a certain channel spacing: (**a**) d/λ=0.2; (**b**) d/λ=0.5.

After experimental verification of the directivity of a sensing channel, the dependence of directivity on DFOH structure is experimentally verified. In signal processing, d/λ is selected as 0.2 m. Directivity functions of the DFOH in the case of L/λ = 1, 1.5, and 2 are calculated as the sum of the outputs phase of all the sensing channels and are presented in Figure 6a. The results in the figure show that as L/λ increases from 1 to 2, the widths of the main lobes decrease from 14.6° to 10.4°. In addition, the height of the side lobes at the same angle *θ* decreases as L/λ increases. The conclusions mentioned above are also valid in the cases of d/λ = 0.5, which are confirmed by the calculated results shown in Figure 6b.

**Figure 7:**
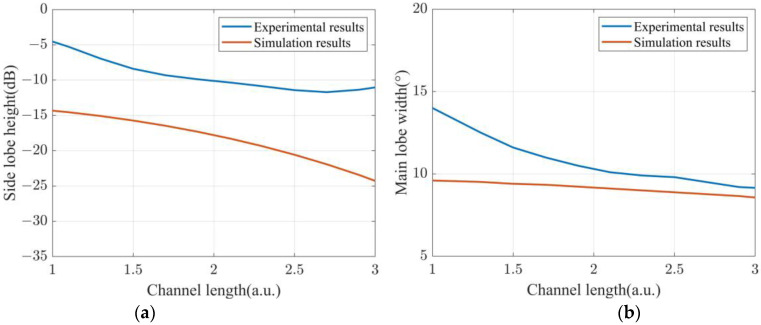
Experimental and simulation results of (**a**) side lobe height and (**b**) main lobe width as a function of channel length.

To specifically verify the dependence of directivity function *B* on the sensing length, the height of the first-order side lobe and the width of main lobe are calculated using the experimental results in the cases of L/λ increasing from 1 to 3 in the step of 0.2. Figure 7a presents the calculated side lobe height as a function of L/λ in the case of d/λ = 0.2. Also presented in Figure 7a are the simulation results of the side lobe height calculated using Equation (4). Both experimental results and simulation results in Figure 7a show a downtrend. Specifically, as the L/λ increased from 1 to 3, experimental results decrease from −5 dB to −11 dB, and the simulation results are reduced from −14 dB to −24 dB. Due to the noise in the experimental environment, the height of the side lobe obtained in the experiment is higher than that in the simulation.

Figure 7b shows the width of the main lobe obtained both in experiments and in simulations, showing a downtrend as L/λ increases. Specifically, the width of the main lobe in the experiment becomes narrower from 14° to 9.1°, and the width of the main lobe in the simulation decreases from 9.5° to 8.6°. The noise in the experiment causes the width of the main lobe to be larger than that in the simulation.

**Figure 8:**
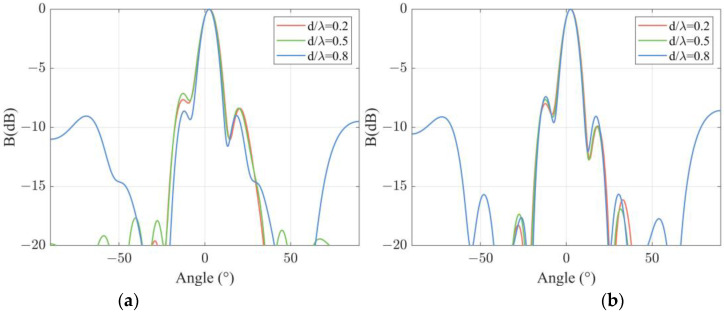
Experimental results of beam patterns at different channel spacings at a certain channel length: (**a**) L/λ=1.5; (**b**) L/λ=2.

The effect of channel spacing on the directivity function of a DFOH is also experimentally studied. The array length is set at 8 m, and L/λ is selected as 1.5. Directivity functions of the DFOH in the case of d/λ = 0.2, 0.5, and 0.8 are calculated and are presented in Figure 8a. The results in the figure show that as d/λ increases from 0.2 to 0.8, the widths of the main lobe decrease from 11.6° to 10.4°. In addition, the heights of the first-order side lobe decrease from −8.39 dB to −9.04 dB. Both the widths of the main lobes and the height of the first-order side lobes change slightly. The conclusions mentioned above are also valid in the cases of L/λ=2, which are confirmed by the calculated results shown in Figure 8b.

**Figure 9:**
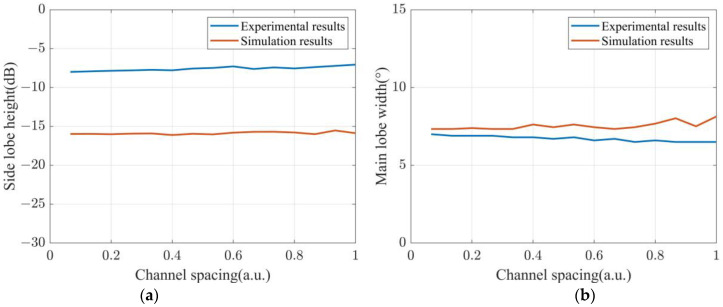
Experimental and simulation results of (**a**) side lobe height and (**b**) main lobe width as a function of channel spacing.

The dependence of directivity function *B* on the sensing spacing is specifically verified. The d/λ is set from 0 to 1 in the step of 0.02. The L/λ is selected as 2. The first-order side lobe height and main lobe width are calculated using the experimental results and are presented in Figure 9. The calculated first-order side lobe height presented in Figure 9a is stable at about −8 dB, and its maximum value is only 0.94 dB higher than the minimum value. Figure 9b presents the calculated main lobe width. The main lobe width is stable at around 7°, and the gap between its maximum value and minimum value is 0.5°. Also presented in Figure 9 are the simulation results of fist-order side lobe height and main lobe width. The simulation results show the same stable trend as the experimental results. However, the noise in the experiment causes different specific values between the simulation results and the experimental results.

## 5. Discussion

Both the simulation results and the experimental results show that the directivity of a DFOH is related to the channel length but not to the channel spacing. Essentially, the directivity of a DFOH is partially governed by the directivity of sensing channels. As stated in Section 2, the directivity of the sensing channel becomes more obvious as the channel length increases, resulting in a more obvious directivity of the DFOH, such as narrower width of the main lobe and lower height of the first side lobe. However, the directivity of the sensing channel is independent of the channel spacing, so the change of the channel spacing has no effect on the directivity of the DFOH.

To realize optimal performance of a DFOH, two steps are expected. Considering the inherent advantage of a DFOH in terms of arbitrary structure design, one is supposed to step firstly to make clear the directivity dependence of a distributed fiber optic hydrophone on array structure, which is the goal of this paper. To further optimize the array beamforming of a DFOH, effort on array signal processing is expected in the second step. For example, the influence of weighting functions (such as Dolph–Chebyshev weighting) on the directivity pattern of a DFOH is expected to be studied. Another example is to introduce a super-directivity method (such as minimum variance distortionless response and deconvolved conventional beamforming) to the array signal processing of a DFOH. The second step is essential and will be investigated in future work.

## 6. Conclusions

In this paper, the dependence of the directivity of a distributed fiber optic hydrophone on array structure is theoretically and experimentally studied. The directivity function of a channel *B*_1_ is obtained by analysis of the output phase of a sensing channel in a distributed fiber optic hydrophone, and the directivity function of a distributed fiber optic hydrophone is derived by adding up the output phase of all the sensing channels. Based on the directivity function, simulations are performed, and the results reveal that the longer sensing channel length guarantees lower first-order side lobe and narrower main lobe of *B*. In addition, the simulation results indicate that the channel spacing is irrelevant to *B* as long as the spacing is shorter than the wavelength of the detected acoustic signal. Experiments are performed, and the results confirm the theoretical analysis. This study would provide guidance for the structure design of a distributed fiber optic hydrophone in signal processing and promote the distributed fiber optic hydrophone in the field of sound orientation.

## Figures and Tables

**Table 1:** Main lobe widths (°) and first side lobe heights (dB) in the case of d/λ=0.25.

L/λ	0.25	0.75	1	6
Main lobe width	5.04	5.04	5.21	4.41
First side lobe height	−13.26	−13.41	−13.54	−25.31

**Table 2:** Main lobe widths (°) and first side lobe heights (dB) in the case of d/λ=0.75.

L/λ	0.25	0.75	1	6
Main lobe width	5.21	5.21	5.16	4.47
First side lobe height	−13.11	−13.26	−13.40	−25.93

**Table 3:** Main lobe widths (°) and first side lobe heights (dB) in the case of L/λ=0.25.

d/λ	0.25	0.5	0.75
Main lobe width	5.04	5.04	5.21
First side lobe height	−13.26	−13.21	−13.11

**Table 4:** Main lobe widths (°) and first side lobe heights (dB) in the case of L/λ=6.

d/λ	0.25	0.5	0.75
Main lobe width	4.41	4.41	4.47
First side lobe height	−25.31	−25.27	−25.93

## Data Availability

Not applicable.

## Appendix A

The detailed procedure for obtaining Equation (3) is given by

(A1) $$\begin{array}{cc} \Delta \phi & =2\int_{x-s}^{x+\left(L-s\right)}\frac{2\pi }{\lambda_{l}}P\eta \cos\left(\frac{2\pi }{\lambda }\sin\left(\theta \right)x-\omega t\right)dx\\ & =\frac{4\pi }{\lambda_{l}}P\eta \frac{\lambda }{\sin\left(\theta \right)2\pi }\left[\sin\left(\frac{2\pi }{\lambda }\sin\left(\theta \right)\left(x+L-s\right)-\omega t\right)-\sin\left(\frac{2\pi }{\lambda }\sin\left(\theta \right)\left(x-s\right)-\omega t\right)\right]\\ & =\frac{4\pi P\eta L}{\lambda_{l}}\sin c\left(\frac{\sin\left(\theta \right)L}{\lambda }\right)\cos\left[\frac{2\pi }{\lambda }\sin\left(\theta \right)x+\frac{2\pi }{\lambda }\sin\left(\theta \right)\left(0.5L-s\right)-\omega t\right]\\ & =\Delta \phi_{0}\cos\left[\frac{2\pi }{\lambda }\sin\left(\theta \right)x+\varphi_{i}-\omega t\right] \end{array}$$
